# Machine learning approach to student performance prediction of online learning

**DOI:** 10.1371/journal.pone.0299018

**Published:** 2025-01-14

**Authors:** Jing Wang, Yun Yu

**Affiliations:** 1 Jiangsu College of Finance and Accounting, Jiangsu, China; 2 Nanjing University of Science and Technology, Jiangsu, China; Virtual University of Pakistan, PAKISTAN

## Abstract

Student performance is crucial for addressing learning process problems and is also an important factor in measuring learning outcomes. The ability to improve educational systems using data knowledge has driven the development of the field of educational data mining research. Here, this paper proposes a machine learning method for the prediction of student performance based on online learning. The critical thought is that eleven learning behavioral indicators are constructed according to online learning process, following that, through analyzing the correlation between the eleven learning behavioral indicators and the scores obtained by students online learning, we filter out those learning behavioral indicators that are weakly correlated with student scores, meanwhile, retain these learning behavior indicators being strongly correlated with student scores, which are used as the eigenvalue indicators. Finally, using the eigenvalue indicators to train the proposed logistic regress model with Taylor expansion. Experimental results show that the proposed logistic regress model defeats against the comparative models in prediction ability. Results also indicate that there is a significant dependency between students’ initiative in learning and learning duration, nevertheless, learning duration has a significant effect on the prediction of student performance.

## I Introduction

Recently, the research being interested in learning behavioral analysis has become increasingly strong. Predicting student performance through online learning analysis can visualize student behavior [1–3], to assist teachers understand the trends in student learning behavior, and to improve curriculum design and teaching quality.

Higher education institutions consider students’ academic performance as one of the most important issues in providing high-quality education to students [4]. Understanding the important factors that influence student performance is complex. Currently, the academic community has used various effective tools and approaches to address student performance challenges [5–7]. In recent years, along with the continuous progress of technology in predicting student performance, there is still a gap to be filled to utilize machine learning and data mining methods to analyze and improve the accuracy of student performance. Many researchers have identified the factors that affect student performance [8]. However, compared to the final student score in the final exam [9], the most common factors depend on learning activities [10]. Therefore, we observe that predicting trends in student performance may be one of the solutions to improve student performance [11].

The Education Data Mining (EDM) method is a solution that may have a potential impact on supporting higher education managers in making data-driven decisions. EDM aims at utilizing new capabilities in data processing and the maturity of data mining algorithms to enhance the learning process and transform existing information into knowledge [12]. EDM analyzes educational data (such as student information, educational records, exam scores, participation in online activities and classroom records, etc.) to develop models to improve learning experiences and institutional effectiveness [13]. Since EDM must discover knowledge from data stored in different formats and granularity levels from multiple sources (such as enrollment systems, registration systems, learning management systems, etc.), each issue requires specific handling. Traditional data mining techniques cannot handle these issues, therefore, the knowledge discovery process requires more advanced data mining methods. EDM applies data mining, statistical methods, and machine learning (decision trees, neural networks, naive Bayes, K-nearest neighbors, etc.) to explore large-scale data generated by educational organizations, in order to better understand the ongoing process.

Researchers have applied EDM methods to curriculum planning and student enrollment prediction [14], enhancing the understanding of the learning process, and examining the chances of course success. Various EDM methods have been applied to forecast students’ behavior [15], which provide feedback and suggestions to students [16] and determine students’ profile in self-regulated learning [17, 18]. The EDM method helps teachers identify students at risk and develop corrective strategies to reduce dropout rates [19, 20] and improve students’ graduation rates [21]. The goal of all these studies is to improve students’ performance. Because of this, most research in this field is focused on modeling students’ performance prediction [22, 23].

Discovering hidden patterns and predicting trends in a large scale of the data might be a potential method to be beneficial for the field of the education [24]. Predictive analysis has been used to address several educational fields, including student performance, dropout prediction, academic warning systems, and course selection [25]. In addition, predictive analysis applying in predicting student performance has increased in recent years [26].

### Motivation

The prediction of student performance can assist students to improve their grades. Many previous studies have proposed that machine learning approaches show potential ascendency in the prediction of student performance. However, for the prediction of student performance, it is difficult to find relevant work on mechanisms to associate the student performance with learning behavior [27]. Hence, the research goal in this work is to forecast student performance according to online learning behavior, and then to analyze the relations between student performance and learning behavior. To finish the goal, here, we firstly constructed eleven learning behavioral indicators based on the online learning process. Through analyzing the correlations between these learning behavioral indicators and the scores obtained by students online learning, we found out learning behavioral indicators that are strong correlated with the scores, following that, the eigenvalue set consists of these learning behavioral indicators that are strong correlated with the scores. Finally, the proposed logistic regress model was trained by the eigenvalue set. Using the trained well logistic regress model to predict student performance.

### Contributions

We summarized the main contributions in this work. As follows

(1) We obtain eigenvalue indicators from the constructed learning behavioral indicators through analyzing the correlation of the both, and then proposed a logistic regress model with Taylor expansion. Using the eigenvalue indicators to train the logistic regress model for the prediction of student performance, instead of using the original data to train it.
(2) We find that there is a significant dependency between students’ initiative in learning and learning duration. However, learning duration has a significant effect on the prediction of student performance.

This paper is arranged as follows. We summarized the related works in Section II. The learning behavioral indicators were constructed and the logistic regress model was proposed in Section III. Experimental settings and results were described in Section IV and Section V, respectively. Section VI discussed the results, and Section VII drew a conclusion and directs future work.

## II Related work

Some efforts regarding the prediction of student performance have been gain, for instance, Conijn et al. [28] predict student performance by using multi-level and standard regressions. Whereas, due to the differences between course data, it is difficult to draw broad conclusions about the online behavior of students with potential risks. The [29] proposed a convolutional neural network for predicting student performance, which of the results show that the prediction is successful. This work utilizes traditional and simple features to establish a student performance prediction model for the prediction of student performance. Similarly, the machined learning method implemented in [30]. Maurya et al. [31] provide a supervised machine learning classifiers and Asalm et al. [32] apply a deep learning model for student performance prediction. But the deep learning model is solely tested on two datasets. And the deep learning model implemented by the [7]. Due to the ability to provide accurate and reliable results, deep learning approaches have become a popular strategy for predicting student behavior. Such as, the method proposed in [33–37].

Logistic regress is called cost function, which uses logical functions as representations of mathematical models. This model performs good contextual analysis on classified data to understand the relationships between variables [38]. For example, a mixed regress model [39] is proposed to optimize the accuracy of student performance prediction, which can predict qualitative values of various factors related to the obtained student grades. However, it is hard to confirm the reliability of the model. Ahmed et al. [40] proposed an approach using regress thought to predict student performance. Together, several single classification algorithms are employed as base classifiers [41], so as to improve the prediction of student performance. However, the base classifiers need to implement optimization techniques to search parameters and configuration in the classification algorithms. Similarly, the logistic regress model in [42].

Additionally, machine learning approaches effectively forecast student performance. For instance, Moreno et al. [43] utilized Waston machine learning approach to derive predictors for the forecast of student’s final grades based on online university setting. The predictors focus on investigating student performance. Qunn et al. [44] employed a learning model using Moodle data to forecast student’s academic performance for a blended education setting, thus forecasting that whether the student would pass or fail in academic examination. The forecast accuracy to the learning model reaches 92.2% in forecasting academic grade of students. Due to relying on Moodle data, using Moodle logs in the previous ten weeks can forecast those failing students, accurately, but unfortunately, using those in the first six weeks to suffer frustration in prediction. Similarly, the [45] utilized Moodle data to the prediction of student performance. Qiu et al [46] developed the behavior classification-based e-learning performance (BCEP) machine learning model to achieve the prediction of student’s learning performance. BCEP model shows significant ascendency in forecasting because of combining feature fusion with behavioral data, however, BCEP model relied on empirical values. Certainly, also including the decision tree implemented in [47], and support vector machine model in [48] and Naïve Bayes model in [48], etc.

## III Methodology

### A. Overall scheme

**Fig 1:**
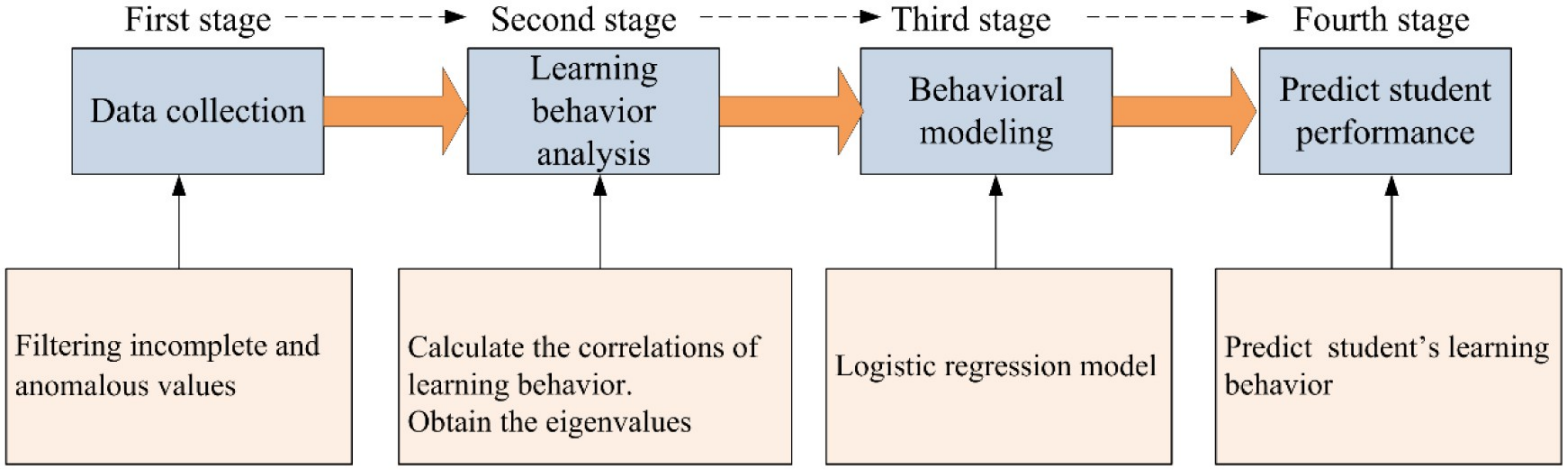
Graphical illustrations of the method. The process contains four stages.

Fig 1 unveils the overall scheme of the method, which involves four stages. In first stage, i.e., data collection. Our goal is the prediction of student performance based on online learning, therefore, the data is collected from online learning platform. The collected data is original from diverse type on the online learning platform, such as relational type and non-relational type, moreover, the data might hide incomplete and anomalous values. Consequently, there must pretreat the data based on the learning behavior indicators constructed by us. By doing so, it can create a condition for the performing of the second stage.

Learning behavior analysis, i.e., the second stage, which classifies students according to certain standards. The purpose of this stage is to compare the learning behaviors for different types of students, thus analyzing their behavior characteristics. To identify whether behavioral indicators are related to the outcome, we analyzed the correlation between learning behavioral indicators and online learning. If the analysis results are no correlation, the behavioral indicators are discarded. Instead, if they are relevant, the behavioral indicators are retained as the eigenvalues.

The task in the third stage is behavior modeling. We constructed a logistic regress model, which is trained by the eigenvalues. The fourth stage is the prediction of student performance using our model.

### B. Analysis of learning behavior

Online learning shows multiple diversity, that is, behavior indicators based on online learning are multiple dimensions. Based on this, we took account into eleven learning behavior indicators, illustrated in Table 1. These learning behavior indicators are described as follows.

**Table 1:** Behavior indicators for online learning.

Learning process	Illustrations	Indicators	ID
Preparation stage	Number of views	Course introduction	CI-N
	Number of registers	Course register	CR-N
	Number of attendances	Course login	CL-N
Major learning behavior	Monitor the learning time	Resource monitoring time	RM-T
	Resource utilization	Resource utilization efficiency	RU-E
	Number of repeated views for resource	Repeated views of resources	RV-N
	Number of repeated learning after finishing course	Repeated learning of resources	RL-N
	Degree of duplicate monitoring of resources	Resource density utilization	RD-U
Secondary learning behavior	Number of forum browse	Forum browsing	FB-N
	Number of forum post	Forum posting	FP-N
	Number of forum reply	Forum replying	FR-N

The learning process consists of three parts in this work, i.e., preparation stage, major learning behavior and secondary learning behavior. In preparation stage, we took account into the number of viewing course introduction, that of course register and that of course login. Following that, major learning behavior and secondary learning behavior are constructed, among them, major learning behavior, which is treated as a critical monitoring factor, consists of the five behavior indicators, including the monitoring of learning time for students, denoted as RM-T, and resource utilization, namely RU-E, which is calculated through the time spent by students on learning resources divided by the total time spent on learning resources (recommended time). As well as, the number of repeated viewing resource, namely RV-N, and the number of repeated learning after finishing course, i.e., RL-N. Resource density utilization, i.e., RD-U, refers to the time of view resource divided by the time difference between last view resource and first view, reflecting students’ concentration. As for the secondary learning behavior, it is regarded as a learning interaction behavior, containing the number of browsing learning forum FB-N, that of posting learning forum FP-N and that of replying learning forum FR-N.

We analyzed the correlation between the eleven learning behavioral indicators in Table 1 and the course of average score achieved by students in Table 2, through using the SPSS tools. Following that, we filter out those learning behavioral indicators with weak correlation, instead, those with higher correlation should be retained. The filtered details are that those learning behavioral indicators with the correlation coefficient below 0.6 are filtered out, otherwise, they are retained. Finally, those retained learning behavioral indicators are used as the eigenvalue indicators affecting online learning. For the convenience of description, in subsequent sections, we denote those retained learning behavioral indicators as the eigenvalue indicators.

**Table 2:** Dataset descriptions.

#	Course name	Requirement learning time	Learning unit quantity	Score for a learning unit	Average score	Learning flag
C1	Operation System	60 hours	10			
C2	Java Programing	60 hours	10			
C3	Python Programing	60 hours	10			
C4	C++ Programing	60 hours	10			
C5	C Programing	60 hours	10			
C6	C# Programing	60 hours	10			
C7	Probability Theory	30 hours	6			
C8	Graph Theory	30 hours	6			
C9	Calculus	30 hours	6			
C10	Optimal Theory	30 hours	6			
C11	Linear Algebra	30 hours	6			
C12	Software Engineering	40 hours	8			
C13	Computer Network	40 hours	8			
C14	Artificial Intelligence	40 hours	8			
C15	Business English	48 hours	12			

### C. Behavioral modeling

We construct a logistic regress model according to the obtained eigenvalue indicators. Having

$$h(x)=g(\theta_{1}x_{1}+\theta_{2}x_{2}+\ldots +\theta_{i}x_{i}+\ldots +\theta_{n}x_{n})=g(\theta^{T}x)$$ (1)

Where *x*_1_, *x*_2_,…, *x*_*i*_,…, *x*_*n*_ is the eigenvalue indicators. *θ*_1_, *θ*_2_,…, *θ*_*i*_,…, *θ*_*n*_ is the weight corresponding to the eigenvalue indicators. The value of *h*(*x*) denotes the probability of taking 1. The joint density function of *n* samples can be calculated, having

$$L(\theta |x,y)=\Pi_{i=1}^{n}{\left(h(x)\right)}^{y(i)}{\left(1-h(x)\right)}^{1-y(i)}$$ (2)

To predict accurately the results, we introduced penalized log-likelihood. That is, Eq (2) is converted into Eq (3). As follows

$$L^{*}=L(\theta |x,y)-\frac{\lambda }{2}\sum_{j=1}^{m}\beta_{j}^{2}$$ (3)

To simplify, taking the logarithm of Eq (3), as follows

$$L^{*}=\sum_{i=1}^{n}y_{i}\;\text{log}\;p_{i}+\sum_{i=1}^{n}\left(1-y_{i})\text{log}(1-p_{i}\right)-\frac{\lambda }{2}\sum_{j=1}^{m}\beta_{j}^{2}$$ (4)

Where *λ* is the penalty item. The larger the values of *λ* are, the stronger the effects are. *y*_*i*_ is the *i*-th eigenvalue indicator. *p*_*i*_ is the probability that *y*_*i*_ = 1. *β*_1_, *β*_2_,…, *β*_*i*_,…, *β*_*m*_ are parameters which could be estimated by maximum likelihood criterion. For the estimate of *β*_*i*_, we referred the [49]. According to Eq (4), we can obtain the predictor, as follows

$$\Theta =\text{log}\frac{p_{i}}{1-p_{i}}$$ (5)

To solve the *p*_*i*_, let **y** = [*y*_1_, *y*_2_,.., *y*_*n*_]^*T*^, **p** = [*p*_1_, *p*_2_,.., *p*_*n*_]^*T*^, *β* = [*β*_1_, *β*_2_,…, *β*_*i*_,…, *β*_*m*_]^*T*^ and let us take the derivative of L* with respect to *β*_*i*_. Having that

$$\partial L*/\partial \beta =0\Rightarrow {\text{y}}^{T}(\text{y}-\text{p})=\lambda \beta$$ (6)

Obviously, Eq (6) is non-linear due to the non-linear relations between **p** and *β*. To obtain a linear equation, let us take the first order Taylor expansion at *p*_*i*_.

$$p_{i}\approx {\tilde{p}}_{i}+\sum_{j=1}^{m}\frac{\partial p_{i}}{\partial \beta_{j}}(\beta_{j}-{\tilde{\beta }}_{j})$$ (7)

Where ${\tilde{p}}_{i}$ and ${\tilde{\beta }}_{j}$ are an approximate solution. For the ${\tilde{p}}_{i}$, we use Eq (8) to calculate, having that

$${\tilde{p}}_{i}=\sum_{j=1}^{n}\beta_{j}x_{i}$$ (8)

At the beginning of iterations, we can initialize a starting value about ${\tilde{\beta }}_{j}$ according to *β*_*i*_. Hence, the value of *p*_*i*_ can be calculated.

### D. Algorithm implementation

The model algorithm is as shown in Algorithm 1. The input and output are the eleven learning behavioral indicators LBI(*k*), the average score of *j*-th course AS(*j*). Firstly, parameters are initialized in Step 1. Following that, the procedure between Step 2 and Step 12 displays the selection of the eigenvalue indicators. Through calculating correlation coefficient *CR*(*k*,*j*) between LBI(*k*) and AS(*j*), we can obtain eigenvalue indicator $\text{LBI(}\hat{k}\text{)}$, illustrated in Step 4 to Step 7. After successfully obtaining eigenvalue indicator $\text{LBI(}\hat{k}\text{)}$, in Step 13, we utilize $\text{LBI(}\hat{k}\text{)}$ construct a matric $M(300,\hat{k}\times 15)$ with 300 rows and $\hat{k}\times 15$ columns. Here, in the matric $M(300,\hat{k}\times 15)$, the row is the number of students, and the column is constructed by both the number of eigenvalue indicators and that of courses. In Step 14 and Step 15, the matric $M(300,\hat{k}\times 15)$ is randomly divided into training set *Train*(*M*) and testing set *Test*(*M*). The procedure between Step 16 and Step 23 illustrates the training of logistic regress model *h*(*x*). Using training set *Train*(*M*) to train *h*(*x*), once maximum training accuracy is obtained, the training is terminated. Meanwhile, current trained logistic regress model *h*(*x*, *Train*(*M*)). Finally, using testing data *Test*(*M*) to verify the trained *h*(*x*, *Train*(*M*)), prediction accuracy is outputted, illustrated in Step 24 and Step 25.

Algorithm 1. Model algorithm

input LBI(*k*), AS(*j*)

output Prediction accuracy

1 Initialize parameter;

2 **For** *k* = 1 **to** 11:

3  **For** *j* = 1 **to** 15:

4   Calculating the correlation between LBI(*k*) and AS(*j*);

5   Obtaining the correlation coefficient *CR*(*k*,*j*);

6   **If** *CR*(*k*, *j*) > = 0.6 **Then**:

7    saving eigenvalue indicator $\text{LBI(}\hat{k}\text{)}$;

8   **End If**

9   *j*++;

10  **End For**

11  *k*++;

12 **End For**

13 Using $\text{LBI(}\hat{k}\text{)}$ to construct a matric $M(300,\hat{k}\times 15)$ with 300 rows and $\hat{k}\times 15$ columns;

14 Obtaining training set *Train*(*M*) through randomly selecting 80% $M(300,\hat{k}\times 15)$;

15 Obtaining testing set $Test(M)=M(300,\hat{k}\times 15)-80\%*M(300,\hat{k}\times 15)$;

16 **For** *i* = 1 **to** i = *I*_*max*_:

17  Using training set *Train*(*M*) to train logistic regress model *h*(*x*) in Eq (1);

18  **If** current training accuracy = = maximum value **True**:

19   Saving the model *h*(*x*, *Train*(*M*));

20   Obtain current training accuracy;

21   **Break**;

22  **End If**

23 **End For**

24 Using testing set *Test*(*M*) to verify *h*(*x*, *Train*(*M*));

25 Obtaining forecasted accuracy;

## IV Experimental settings

### A. Datasets

Experimental datasets are provided from the MOOC platform (https://www.icourse163.org/). The datasets include 15 courses. We manually collected information for 15 courses, and the course is lasted for 10 weeks, among them, 300 students enrolled in the course. We used the average grade to characterize student’s performance, that is, we set the score to be excellent or not as the dependent variable. If the score is greater than 90 points, it is treated as excellent. Otherwise, it is treated as not excellent. The Table 2 lists the details of the 15 courses, where requirement learning time refers to the sum time fulfilling the corresponding course. Learning unit quantity is the number of learning units included in a course. Score for a learning unit indicates the testing grade obtained by a student after fulfilling a learning unit. Average score is the average value of the testing grade for all learning units. Learning flag includes two metrics *Ex* and *NE*, if average score is greater than 90 points, learning flag is marked with *Ex*, otherwise, it is marked with *NE*. The dataset is illustrated in Table 2.

### B. Competitors and evaluated indicators

Apart from our logistic regress model, the logistic model [28], the mixed regress model [39], the logistic model [42], decision tree model [47] and SVM model [48] are used as a comparison. We chose the five comparative models to have a fair comparison. Please note that our model and the five competitors, the same eigenvalues are used as the training set and testing set.

In addition, the metrics Accuracy, Precision, Recall and F1-score are used to evaluate the predicted ability of these methods. We utilized the Python language to achieve the four algorithms corresponding to the four models, unless other stating, the four algorithms were run on the same experimental setting.

### C. Experimental designs

We conducted three groups of experiments to verify the proposed method. As follows,

Experiment (I). Eigenvalue indicators selection. To obtain critical indicators impacting student grade, we screened the eleven learning behavioral indicators by using correlation analysis and clustering methods. Then, the results were compared.
Experiment (II). Comparisons of prediction performance. To verify the proposed logistical regress model, we compared it with the five opponents on the eigenvalue indicators. Then, the compared results were analyzed.
Experiment (III). Learning efficiency. To analyze the learning efficiency online learning, we calculated the learning scores of 300 students for viewing courses on 10 weeks. Then, the calculated results were analyzed.

## V Results analysis

This section presents the experimental results. We discussed the result analysis from three aspects, including eigenvalue selection, the prediction for student performance and students’ enthusiasm for active learning. The details are as follow.

### A. Selections of eigenvalue indicators

We analyzed the relations between the eleven learning behavior indicators (i.e., the indicators in Table 1) and student scores by SPSS tool, as shown in Table 3. The five Indicators CI-N, CR-N, CL-N, RM-T and RL-N are no significant correlated with the learning scores. The three indicators FB-N, FP-N and FR-N are weakly correlated with the learning scores. While for the three indicators the number of repeated views for resource RV-N, resource utilization efficiency RU-E and resource density utilization RD-U, they have a strong correlation with the learning scores. Consequently, together, the three learning behavioral indicators RV-N, RU-E and RD-U are used as the eigenvalue indicators, which are used to train and test our logistic regress model and the four opponents.

**Table 3:** Correlations of learning behavioral indicators and student scores. Sign ** indicates that there is a significant correlation at 0.05 level (two tailed).

Learning behavior indicators	Pearson correlation	Scores *Sig(two tailed)*	Number of samples
CI-N	0.036	0.302	300
CR-N	0.099	0.314	300
CL-N	0.036	0.322	300
RM-T	0.014	0.181	300
RL-N	0.069	0.283	300
FB-N	0.212	0.040	300
FP-N	0.247	0.032	300
FR-N	0.256	0.033	300
RV-N	0.781 **	0.000	300
RU-E	0.701 **	0.000	300
RD-U	0.708**	0.000	300

**Fig 2:**
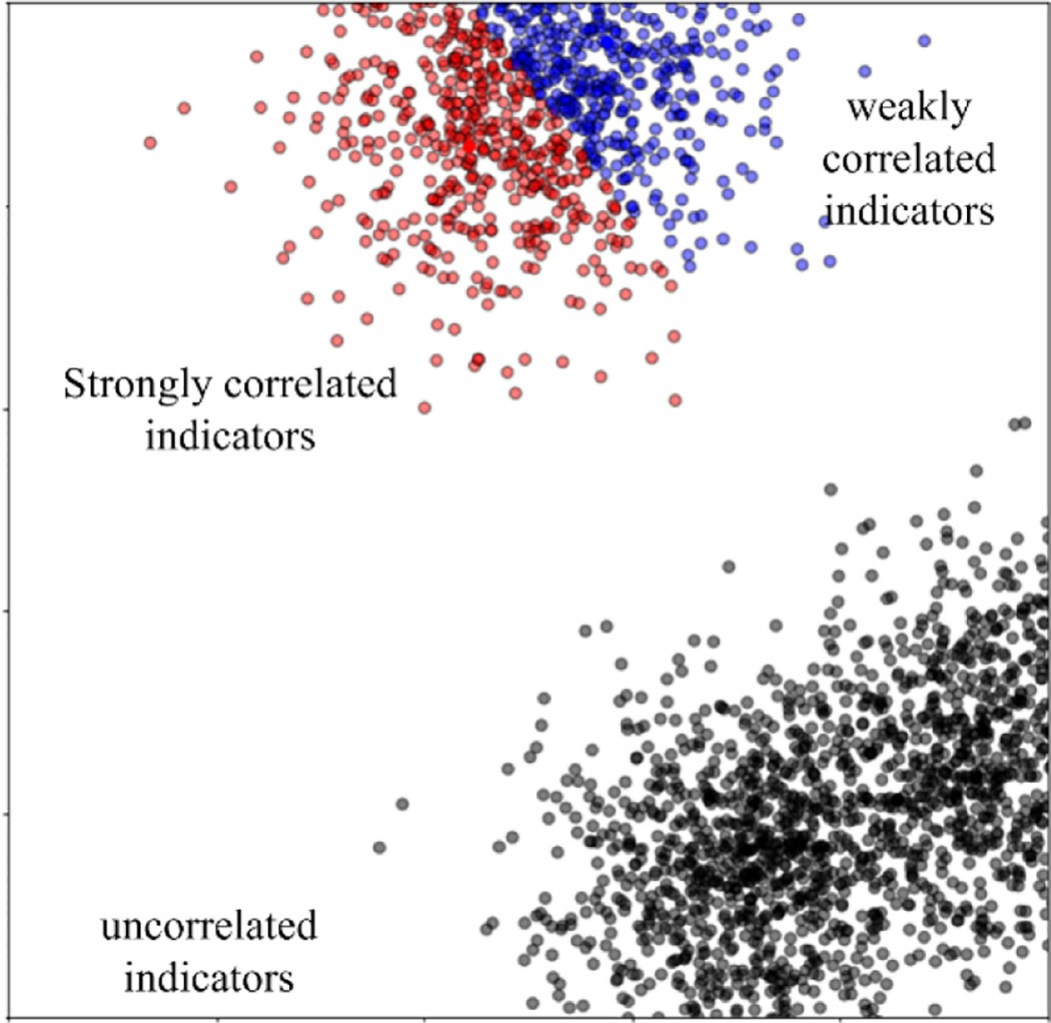
Clustering for the eleven learning behavioral indicators. The three indicators RV-N, RU-E and RD-U are marked with red circles. The three indicators FB-N, FP-N and FR-N are marked with black circles. The five Indicators CI-N, CR-N, CL-N, RM-T and RL-N are marked with green circles.

We clustered the eleven learning behavior indicators by k-means clustering. These values regarding the eleven learning behavior indicators are obtained via the 300 students learned 15 courses on ten weeks. According to the results analyzed by Table 3, the clustered results are shown in Fig 2, where *c* = 3. In Fig 2, the clustering results of the eleven learning behavioral indicators are significant from the perspective of correlations when *c* is equal to 3. Hence, it is reasonable to choose the three learning behavioral indicators RV-N, RU-E, RD-U as the eigenvalue indicators from the view of the correlation.

### B. Comparisons of predication performance

Now, we start to verify the predication ability of our logistic regress model. The eigenvalue set was randomly divided into a training set and a testing set, among them, the 80% of the set was used as the training set, and the rest 20% is testing set. The comparative results are given in Table 4, showing that our logistic regress model defeats against the five opponents. Moreover, our model can predict student performance more accurately.

**Table 4:** Predication results on three eigenvalue indicators. These results are averages of 100 times. The best results are highlighted.

	Training set				Testing set			
	Accuracy	Precision	Recall	F1-score	Accuracy	Precision	Recall	F1-score
Our model	**0.947**	0.928	**0.933**	**0.979**	**0.933**	**0.909**	0.922	**0.966**
Logistic model [28]	0.930	0.922	0.894	0.955	0.912	0.900	0.927	0.873
Mixed regress model [39]	0.942	**0.935**	0.886	0.951	0.903	0.902	0.918	0.918
Logistic model [42]	0.937	0.912	0.872	0.967	0.909	0.903	0.909	0.903
Decision Tree [47]	0.911	0.922	0.909	0.918	0.882	0.906	**0.937**	0.946
SVM [48]	0.907	0.918	0.911	0.887	0.898	0.904	0.871	0.951

The results in Table 4 are based on the eigenvalue indicators consisted of the three learning behavioral indicators RV-N, RU-E and RD-U that are strong correlated with student scores. To objectively assess the model, we supplemented the three learning behavioral indicators FB-N, FP-N and FR-N that are weakly correlated with student scores. Together, the six learning behavioral indicators RV-N, RU-E, RD-U, FB-N, FP-N and FR-N are used as the eigenvalue set, which test the six models.

Table 5 unveils the prediction results, showing that our model is still better than the five competitors in prediction capabilities. Compared these results in Table 5 to those in Table 4, we find that the prediction results of the six models did not show significant changes. Moreover, the changes in the predicted results of using the three learning behavioral indicators and using the six learning behavioral indicators, indicating that although the three learning behavioral indicators FB-N, FP-N and FR-N were supplemented (i.e., six learning behavioral indicators in total), there is a weak influence in predicting the results. Hence, these results confirm each other with the results in Table 3.

**Table 5:** Predication results on six eigenvalue indicators. These results are averages of 100 times. The best results are highlighted.

	Training set				Testing set			
	Accuracy	Precision	Recall	F1-score	Accuracy	Precision	Recall	F1-score
Our model	**0.959**	**0.894**	**0.937**	0.916	**0.933**	**0.901**	**0.917**	**0.959**
Logistic model [28]	0.911	0.838	0.880	**0.944**	0.884	0.900	0.909	0.944
Mixed regress model [39]	0.912	0.847	0.893	0.902	0.869	**0.901**	0.906	0.941
Logistic model [42]	0.909	0.811	0.882	0.917	0.913	0.875	0.914	0.950
Decision Tree [47]	0.922	0.825	0.859	0.916	0.863	**0.901**	0.900	0.952
SVM model [48]	0.847	0.888	0.826	0.833	0.820	0.863	0.913	0.955

### C. Learning efficiency

**Fig 3:**
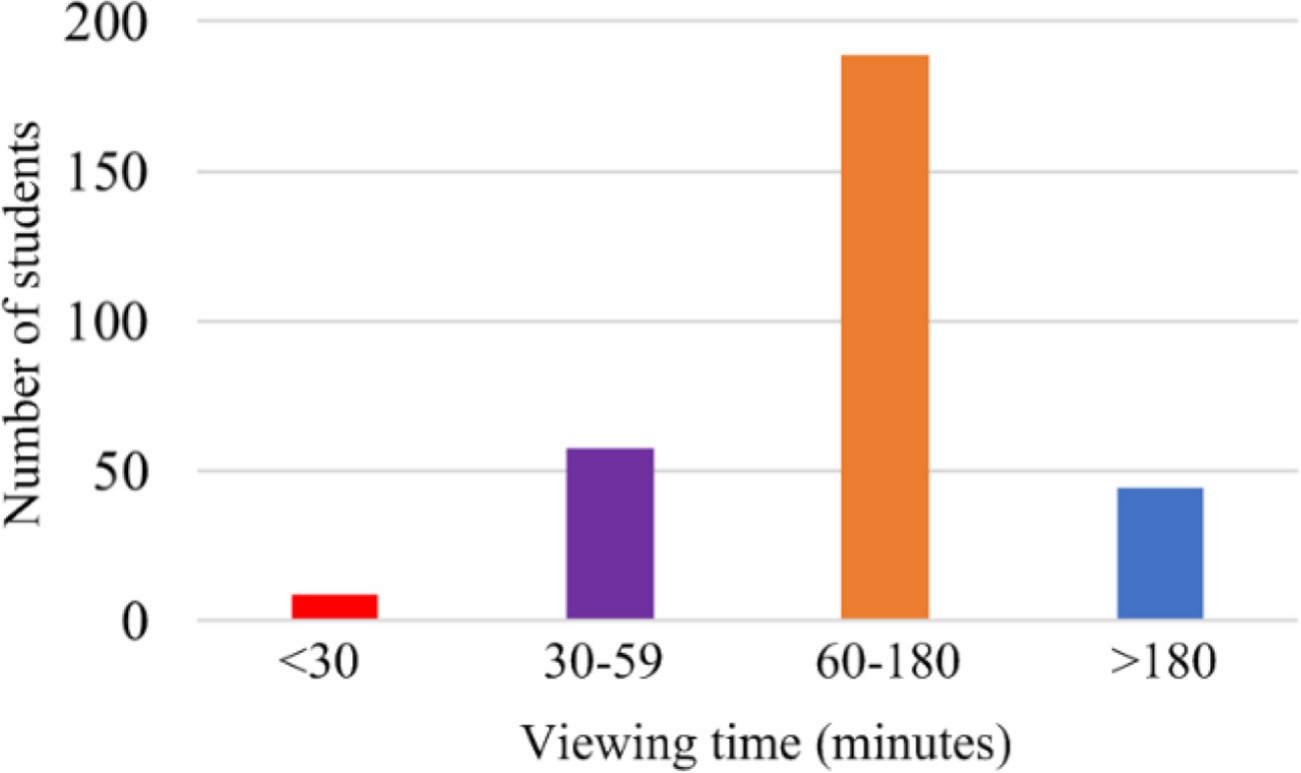
Proportion distribution of viewing course time per week.

Fig 3 unveils average viewing time of a week for the fifteen courses, showing that majority students (i.e., 63.0% students) consume 60 to 180 minutes on the course viewing per week. And 14.9% students prefer to consume more than 180 minutes for the course viewing per week. However, 19.3% students choose 30 to 59 minutes for the course viewing per week. Certainly, rest of 2.8% spend less than 30 minutes for the course viewing per week, or not for the viewing. These indicate that students exhibit certain proactive learning behaviors.

**Fig 4:**
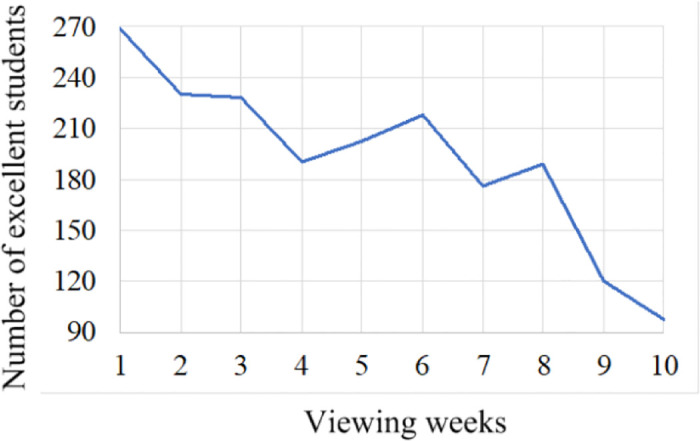
Relations between excellent students and viewing time. The score greater than 90 points is regarded as excellent. Otherwise, not excellent.

Fig 4 unveils the number of excellent students. As the viewing time increase, the number of excellent students shows a slight downward trend. This implies that there is a significant dependency between students’ initiative in the learning and learning duration. Noting that we did not consider the difficulty of the course, i.e., the impact of course difficulty on students’ learning motivation. This is to objectively evaluate students’ initiative in the learning.

## VI Discussions

### A. Advantages

The predictor Θ in Eq (5) have ascendency forecast ability. In the process of deriving the predictor Θ, we took account into the first order Taylor expansion, illustrated in Eq (7). By doing so, the predictor Θ can better approximate the original data. In fact, we sufficiently borrowed that the advantage Taylor expansion can approximate object functions. This is one advantage of our method. Additionally, we did not impose any assumptions on the data distribution and our model, which is another advantage. In summary, that is why our method won.

### B. Limitations

Our goal is the student performance prediction for online learning, therefore, we constructed the eleven learning behavioral indicators. However, many factors have an effect on learning behavioral of students, such as, subjective factors, including students’ emotions. For those subjective factors, in this work, they were not taken, instead, major objective factors were considered. From the perspective of data level, the constructed eleven learning behavioral indicators own limitations. From the view of method level, using the eigenvalues indicators selected from the eleven learning behavioral indicators to train our method, therefore, the construction of the eleven learning behavioral indicators directly influences our forecast results. But please note there are various learning behavioral indicators in real applications, consequently, it is unrealistic to list all those indicators. That is why we chose major learning behavioral indicators in this work.

### C. Insights

Generally, learning behaviors between students exist a difference. Indeed, the characteristics of learning behaviors to students can objectively reflect student performance (regarding the interpretation, please see Section Introduction), therefore, there must be the correlation between the characteristics of learning behaviors and student performance. However, this work used learning behavioral indicators as specific manifestations to the characteristics of learning behaviors. If the impact indicators are different (our eleven learning behavior indicators can be considered as eleven different impact indicators), then their impact on the results may be different. As such, based on this, here, we utilized the strength-weakness of the correlation to explore the latent relationships between learning behavioral indicators and student performance, assisting that we find those relative critical learning behavioral indicators impacting student performance. Moreover, this also help to understand the underlying educational dynamics.

## VII Conclusion

Early prediction of students’ performance is helpful for teachers to determine which students may perform poorly in final examination. Teachers can pay extra attention to those students, meanwhile, take intervened measures to assist them. Timely intervened measures employed by teachers can significantly reduce the number of failed students.

In this work, we constructed eleven learning behavioral indicators aiming at online leaning. Based on the constructed eleven learning behavioral indicators, here proposed a logistic regress model to forecast student performance. The critical thought is that the eigenvalue indicators were chose by calculating the correlations of the eleven learning behavioral indicators and student scores. Following that, the eigenvalue indicators are used to the training set of the proposed logistic regress model. Finally, experimental results show that our model defeats against the comparative models in predicted student performance. We indicate that we did not impose any assumptions on the data distribution and the proposed method, therefore, our method is suitable for the prediction of student performance in complex education environments. We also find that there is a significant dependency between students’ initiative in learning and learning duration. However, learning duration has a significant effect on the prediction of student performance. We demonstrate that the constructed learning behavioral indicators are reasonable, which can provide suggestions to promote students’ enthusiasm for the learning.

Although we accurately predict student performance by the learning behavioral indicators, many factors have an influence on student performance, such as subjective factors in the learning process. Therefore, in future work, we will look at exploring students’ subjective factors impacting student performance.

## Supporting information

S1 Dataset (TXT)
